# Usage of synthetic tendons in tendon reconstruction

**DOI:** 10.1186/1753-6561-9-S3-A68

**Published:** 2015-05-19

**Authors:** Shalimar Abdullah

**Affiliations:** 1 Department of Orthopaedics, Universiti Kebangsaan Malaysia, Kuala Lumpur, 56000 Malaysia

## 

Damaged tendons or loss of tendon poses a major surgical reconstructive problem. The conventional method is harvest donor tendons from palmaris longus, plantaris or tensor fasciae lata. Synthetic tendons has been used in Achilles tendon repair with studies on equine subjects. We present successful use in three patients with extensive tendon loss secondary to polytrauma. Additionally we have conducted an in-vitro study to analyse the ingrowth of tenocytes into the synthetic tendon.

The synthetic tendon utilized in our study is Ortho-tape, a polyethylene terephthalate (polyester) (Neoligaments, Leeds, UK). Its construct is non-absorbable, woven with longitudinal and transverse fibres crossing at right angles. It has an “open structure” acting as a scaffold allowing bone and tissue ingrowth. It is not to be confused with the “permanent type” implant such as Gore-tex which is designed to last a lifetime with no contribution from the host tissue or new tissue growth.

Artificial ligaments clinically available has been carbon (Johnson and Johnson), carbon and polyester (Surgicraft), Leeds-Keio polyester (Neoligaments), Dacron (Stryker-Meadox), bovine glutaraldehyde-fixed xenograft (Xenotech) and Gore-Tex polytetrafluoroethylene (WL Gore).

Synthetic textile implants do not require donor harvesting shortening surgical procedural time and allows retention of initial strength whilst autografts undergo a necrotic stage before eventually recovering over a long period of time. Additionally, donor tendon grafts have varying properties and dimensions with limited availability.

Textile implants has been utilized in ACL reconstruction and patellar ligament reconstruction [1-3] however there has been a decline in usage do to some concerns of synovitis after a prolonged duration 4. Hence some authors recommended usage only in low-activity patients or if donor harvesting was an issue.

## Clinical cases

**Figure 1:**
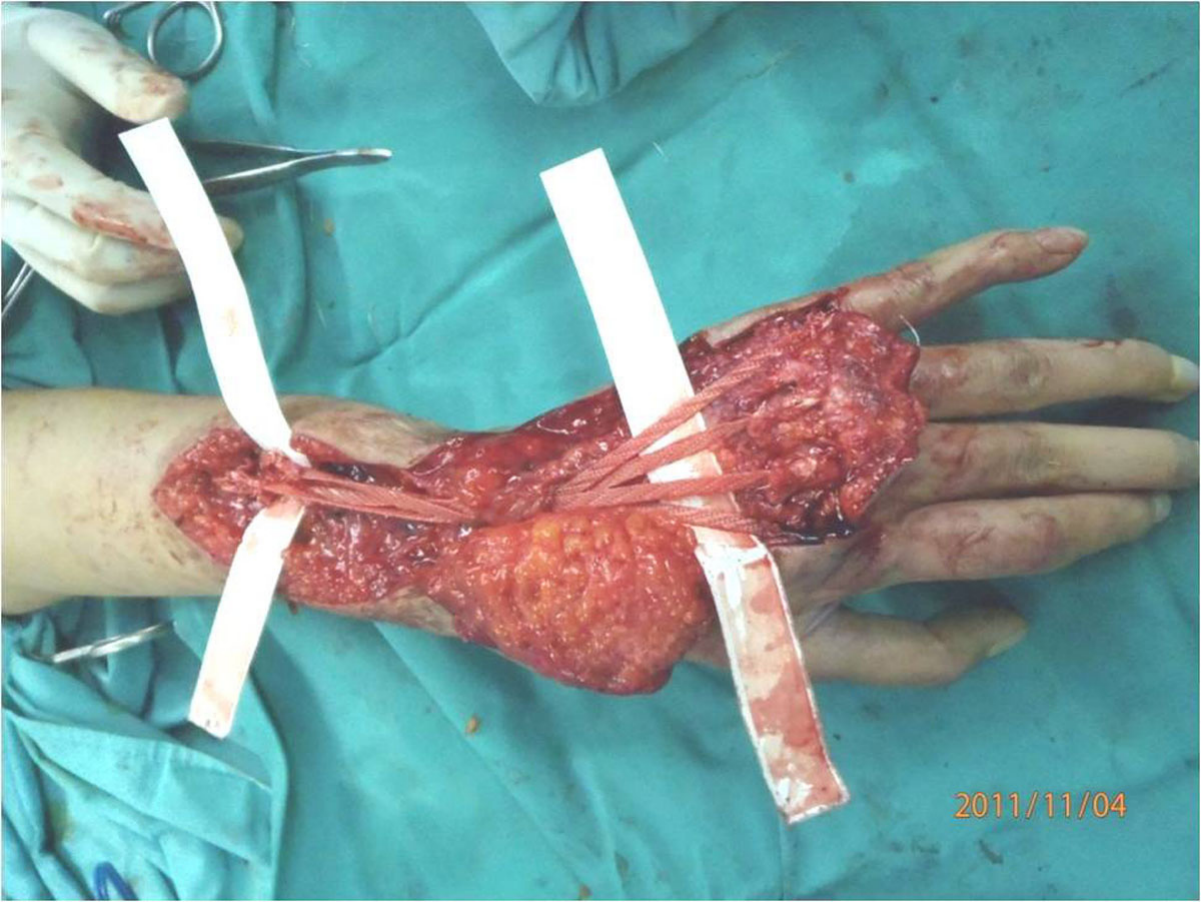
Synthetic textile implant utilized to reconstruct massive extensor tendon loss

Our first patient sustained a degloving wound of the dorsum of her left hand with loss of the proximal half of the 1^st^ to 5^th^ metacarpal bones and distal carpal bones. The EDC to index to small fingers were all absent with a tendon gap of 7cm. After multiple wound debridements, and stabilization with K-wires, she underwent wound coverage with a radial forearm flap from the opposite forearm. Six months later, we proceeded to plate a block of iliac crest bone graft into the empty bony defect. Subsequently after a further six months, we utilized synthetic textile implants to replace the extensor tendon loss (Figure 1).

Initially this was meant to be the first stage of a tendon procedure to be replaced with her own palmaris and plantaris grafts. However her function was good and she declined the second stage. She was able to extend all her fingers fully with a DASH score of 18.8 at 3-years follow-up. Dynamometer grip force was 2kg. Pinch grip was 2.5kg.

Our second patient was a 43-year-old lady who sustained an industrial accident with near total amputation of her right wrist with an open fracture Grade IIIC of her right radius and ulna. Her EDC to her index to little fingers were all avulsed. Her DASH score post-operatively was 58.6. Dynamometer was 0 kg and pinch force was 0kg. She was able to gain extension at the MCPJ of 30 degrees at 3 years follow-up.

In both patients, the Orthotape was measured to length from the tendinous origin of the muscle till the distal remnant of existing tendon. Appropriate tension was utilized before the Orthotape was sutured using the Pulvertaft weave augmented with Prolene 3/0 sutures at both these ends. Any pulleys or retinaculum was reconstructed along the length of the Orthotape. Patients underwent immediate physiotherapy the next day post-operatively.

## In-vitro study

Subsequently we explored seeding a three dimensional tensile scaffold to human tendons of the hand with human tenocyte cells in vitro and incubating it in culture medium for 2,4,6 and 8 weeks. Our methodology consisted of harvesting tendons from patients indicated for upper limb amputation following consent of the patient. Both ends of a 0.5cm strip of synthetic tendon will have a 1cm strip of flexor or extensor tendon sutured to it. The whole specimen will then be immersed and cultured in growth medium. Eight samples were prepared and maintained in a CO2 incubator with a change of growth medium thrice a week.

At the end of 2, 4, 6 and 8 weeks, samples of the tendon from the designated weeks were fixed with formalin overnight, embedded in paraffin and cut from paraffin blocks using a microtome. Haemoxylin and eosin staining were performed. Tissue characteristics and percentage of growth of tenocytes were examined.

Results show no tenocyte growth in the first 2 weeks of incubation. However at 4 weeks, tenocytes were seen migrating towards the junction between the tendon and synthetic scaffold. At 6 weeks, more tenocytes were seen growing into the synthetic scaffold and at 8 weeks, tenocytes were seen in abundance in the synthetic tendon. Thus tenocytes from human tendon are able to gradually proliferate into a synthetic tendon scaffold.

## Conclusion

We recommend usage of synthetic polyester tendon implants as an alternative to tendon autografts in hand reconstruction with massive tendon loss.
